# Preclinical efficacy for a novel tyrosine kinase inhibitor, ArQule 531 against acute myeloid leukemia

**DOI:** 10.1186/s13045-019-0821-7

**Published:** 2020-01-28

**Authors:** Ola A. Elgamal, Abeera Mehmood, Jae Yoon Jeon, Bridget Carmichael, Amy Lehman, Shelley J. Orwick, Jean Truxall, Virginia M. Goettl, Ronni Wasmuth, Minh Tran, Shaneice Mitchell, Rosa Lapalombella, Sudharshan Eathiraj, Brian Schwartz, Kimberly Stegmaier, Sharyn D. Baker, Erin Hertlein, John C. Byrd

**Affiliations:** 1 Division of Hematology, Department of Internal Medicine, Comprehensive Cancer Center, The Ohio State University, Columbus, 455 Wiseman Hall, 400 West 12th Avenue, Columbus, OH 43210 USA; 2 Division of Pharmaceutics and Pharmaceutical Chemistry, The Ohio State University, Columbus, OH USA; 3 Center for Biostatistics, Department of Biomedical Informatics, The Ohio State University, Columbus, OH USA; 4 ArQule, Inc, Burlington, MA USA; 5 Department of Pediatric Oncology, Dana-Farber Cancer Institute, Boston Children’s Hospital, Boston, MA USA

**Keywords:** Acute myeloid leukemia, Multi-kinase inhibitor, ArQule 531

## Abstract

**Background:**

Acute myeloid leukemia (AML) is the most common type of adult leukemia. Several studies have demonstrated that oncogenesis in AML is enhanced by kinase signaling pathways such as Src family kinases (SFK) including Src and Lyn, spleen tyrosine kinase (SYK), and bruton’s tyrosine kinase (BTK). Recently, the multi-kinase inhibitor ArQule 531 (ARQ 531) has demonstrated potent inhibition of SFK and BTK that translated to improved pre-clinical in vivo activity as compared with the irreversible BTK inhibitor ibrutinib in chronic lymphocytic leukemia (CLL) models. Given the superior activity of ARQ 531 in CLL, and recognition that this molecule has a broad kinase inhibition profile, we pursued its application in pre-clinical models of AML.

**Methods:**

The potency of ARQ 531 was examined in vitro using FLT3 wild type and mutated (ITD) AML cell lines and primary samples. The modulation of pro-survival kinases following ARQ 531 treatment was determined using AML cell lines. The effect of SYK expression on ARQ 531 potency was evaluated using a SYK overexpressing cell line (Ba/F3 murine cells) constitutively expressing FLT3-ITD. Finally, the in vivo activity of ARQ 531 was evaluated using MOLM-13 disseminated xenograft model.

**Results:**

Our data demonstrate that ARQ 531 treatment has anti-proliferative activity in vitro and impairs colony formation in AML cell lines and primary AML cells independent of the presence of a *FLT3* ITD mutation. We demonstrate decreased phosphorylation of oncogenic kinases targeted by ARQ 531, including SFK (Tyr416), BTK, and fms-related tyrosine kinase 3 (FLT3), ultimately leading to changes in down-stream targets including SYK, STAT5a, and ERK1/2. Based upon in vitro drug synergy data, we examined ARQ 531 in the MOLM-13 AML xenograft model alone and in combination with venetoclax. Despite ARQ 531 having a less favorable pharmacokinetics profile in rodents, we demonstrate modest single agent in vivo activity and synergy with venetoclax.

**Conclusions:**

Our data support consideration of the application of ARQ 531 in combination trials for AML targeting higher drug concentrations in vivo.

## Introduction

Acute myeloid leukemia (AML), the most common form of acute leukemia in adults, is a rapidly progressing aggressive hematopoietic malignancy arising from the bone marrow myeloid progenitor cells [1] characterized by a differentiation block and aberrant proliferation of these leukemic blasts. AML treatment remains challenging owing to its complex biology, heterogeneity, and the coexistence of multiple genetically aberrant clones within the same patient [1]. In adult patients younger than 60 years of age, the achieved 5-year survival rate is 40–50% while remaining less than 10% for high-risk and elderly patients [2]. The standard therapy for AML has predominantly been 7 + 3 induction cytotoxic chemotherapy with cytarabine and daunuroubicin [3]. However, in the past 2 years, eight drugs were approved by the Federal Drug Administration (FDA) [reviewed in [4]]: Midostaurin [5], CPX-351 [6], gemtuzumab ozogamicin [7], enasidenib [8], ivosidenib [9], gilteritnib [10], venetoclax [11, 12], and glasdegib [13]. Despite the clinical progress of targeted therapies driven by advances in high-throughput sequencing, AML remains difficult to treat and ultimately to cure. AML often develops through the acquisition of multiple somatic mutations with a specific temporal order, where mutations in epigenetic modifying proteins (IDH1, IDH2, TET2, DNMT3A, and ASXL1) or other (TP53, JAK2, SRSF2, and SF3B1) proteins are acquired in the founding clone, while mutations in proteins effecting signaling (FLT3, KIT, PTPN11, N-RAS, KRAS, and CBL) and epigenetic re-programing (NPM1) are secondary mutations acquired during leukemogenesis [14].

*FLT3* mutations, either as an internal tandem duplication (FLT3-ITD) or tyrosine kinase domain point mutation (FLT3-TKD), occur in 25% and 7% of AML, respectively, and constitutively activate the FLT3 proliferation and cell survival pathway [3, 14]. FLT3-ITD mutations are associated with poor prognosis [15], increased relapse, and lower overall survival [16]. The prominence of *FLT3* mutations in AML patients has prompted the development of FLT3 inhibitors such as quizartinib, midostaurin, and gilteritinib. Despite the impressive clinical response with FLT3-ITD selective inhibitor, quizartinib, 50% of patients relapsed within 3 months [17] due to acquired mutations in the TKD activation loop such as D835 and the gatekeeper F691 [18–20]. However, the use of broader kinase inhibitors such as the first-generation multi-kinase inhibitor midostaurin improved overall survival in younger adult patients in combination with intensive chemotherapy [21], and gilteritinib, which inhibits FLT3-ITD, FLT-TKD, and AXL, was demonstrated to induce durable remissions leading to FDA approval for both drugs in newly diagnosed FLT3 mutant AML and relapsed/refractory AML, respectively.

Other kinases have been shown to be relevant to AML and to potentially cooperate with FLT3, such as the SFK [22–26]. Specifically, 76% of primary AML cells have increased Lyn kinase activity [27], and the inhibition of Lyn activity substantially reduced the growth of AML cell lines [24]. Importantly, FLT3-ITD exhibited a higher Lyn binding affinity than FLT3-wild type (FLT3-WT), demonstrating the importance of Lyn in the proliferative FLT3-ITD signal transduction pathway [23]. Likewise, Fyn expression is differentially expressed in AML patient samples [26], and patients with both FLT3-ITD mutations and elevated Fyn expression exhibit inferior survival compared with patients with low Fyn expression [26]. Recently, Marhäll et al. [28] demonstrated the cooperative role of the SFK member LCK in FLT3-ITD oncogenesis via enhancing FLT3-ITD mediated proliferative capacity and STAT5 phosphorylation. Most importantly, targeting SFK disrupts SYK phosphorylation [29], which is upregulated in FLT3-ITD patients and transactivates FLT3 [30, 31]. Finally, several studies have shown the importance of BTK in FLT3 AML pathogenesis [32, 33]. Together, these studies suggest that the use of a multi-kinase inhibitor targeting SFK and BTK could achieve clinical benefit by targeting upstream regulators of FLT3 similar to the use of dual FLT3/SYK inhibitors in AML [31, 34, 35].

ArQule 531 (ARQ 531) is an orally bioavailable kinase inhibitor with favorable pharmacokinetic (PK) properties. In cynomolgus monkeys, ARQ 531 achieved 9 μM maximum concentration within 6 h (*T*_max_) after a single 10 mg/kg oral dose with 72% oral bioavailability [36]. Among other kinases, ARQ 531 has been shown to be a potent and reversible BTK inhibitor and is currently being investigated in a phase 1 trial in patients with relapsed/refractory B cell malignancies (ClinicalTrials.gov Identifier: NCT03162536) [37]. Using in vitro kinase screening, we found that ARQ 531 additionally has inhibitory activity against SFK (Tyr416) [36] and FLT3 suggesting that ARQ 531 has therapeutic potential in AML. In this study, we report the preclinical efficacy of ARQ 531 in AML using in vitro and in vivo models.

## Materials and methods

### Chemical compounds

ARQ 531 was synthesized and provided by ArQule, Inc., (Burlington, MA). Quizartinib, midostaurin, and dasatinib were purchased from LC Laboratories (Woburn, MA). Gilteritinib and venetoclax were purchased from Chemietek (Indianapolis, IN). Entospletinib (GS-9973) was purchased from Selleckchem (Houston, TX). All chemical compounds were dissolved in Dimethyl sulfoxide (DMSO) to obtain a 10 mM stock solution.

### Cell lines and culture conditions

MOLM-13, MV4-11, and OCI-AML3 AML cell lines were purchased from the Leibniz Institute DSMZ-German Collection of Microorganisms and Cell Cultures (Germany). THP-1 and U937 AML cell lines were purchased from the American Type Culture Collection (Manassas, VA). HS-5 GFP were obtained from Dr. William Dalton (H. Lee Moffitt Cancer Center & Research Institute).

All cell lines were cultured in Roswell Park Memorial Institute 1640 medium (Life Technologies, Carlsbad, CA) supplemented with 10% fetal bovine serum (VWR Life Science Seradigm, Radnor, PA), 2 mM L-glutamine, 100 U/mL penicillin, and 100 μg/mL streptomycin (Life Technologies, Carlsbad, CA). The luciferase expressing MOLM-13 cell line was a generous gift from Dr. Ramiro Garzon [38]. Drug-resistant MOLM-13 cells (D835 mutation) and Ba/F3 murine Pro-B cell lines expressing FLT3-ITD or FLT3-TKD or FLT3-ITD-TKD were a generous gift from Dr. Sharyn Baker (College of Pharmacy, OSU) and cultured as previously described in Zimmerman et al. [39]. All cell lines were routinely tested for mycoplasma contamination and authenticated using short tandem repeat (STR) profiling.

### Constitutive SYK overexpressing Ba/F3-FLT3-ITD cell lines

The pMSCV-IRES-Tomato (pMIT-Empty) and pMIT-SYK-TEL vectors were a generous gift from Dr. Kimberly Stegmaier (Dana-Farber Cancer Institute) and previously described in Puissant et al. [30]. HEK293T were transfected with the pMIT vectors and packaging vectors, VSV and GP2. The resulting supernatant was harvested and concentrated for spin infection of Ba/F3 expressing FLT3-ITD (Ba/F3-FLT3-ITD). After recovery, cells were sorted using FACS Aria III flow cytometer.

### Primary patient samples

AML patient samples were obtained from The Ohio State University Comprehensive Cancer Center Leukemia Tissue Bank under an Institutional Review Board-approved protocol.

### Illumina sequencing

For AML cell lines and primary patient samples, genomic DNA was extracted using the DNeasy Blood and Tissue Kit (QIAGEN, Hilden, Germany). The mutational status of 91 protein-coding genes was determined using Illumina TruSeq Custom Amplicon library preparation. Libraries were pooled and sequenced using the MiSeq platform (Illumina, San Diego, CA). DNA library preparations were performed according to the manufacturer’s instructions. Illumina amplicon sequenced reads were aligned to the hg19 genome build using the Illumina Isis Banded Smith-Waterman aligner. Single nucleotide variant and indel calling were performed using MuTect and VarScan, respectively. The MuCor algorithm was used as the baseline for integrative mutation assessment. We only considered non-synonymous variants not listed in either the 1000 Genome database or dbSNP142-common variants. After variant calling, variants were annotated using SnpEff and vcfanno along with the dbsnp, COSMIC, 1000 genomes, and 6500 exomes variant databases. The Mucor3 algorithm was used as the baseline for integrative mutation assessment. All called variants underwent visual inspection of the aligned reads using the Integrative Genomics Viewer.31. Variants in regions of high discrepancy, low quality, tandem repeats, or mononucleotide runs were excluded.

### Antibodies

Antibodies purchased from Cell Signaling Technology (CST, Danvers, MA) including anti-FLT3 (Cat.#3462), anti-phospho-FLT3 Tyr589/591 (Cat.#3464), anti-STAT5 (Cat.#9363), anti-phospho-STAT5 (Cat.#9351), anti-ERK (Cat.#4695), anti-phospho-ERK (Cat.#4377), anti-Src family kinase (Cat.#2109), anti-phospho-Src family kinases Tyr416 (Cat.#2101), anti-SYK (Cat.#13198), anti-phospho-SYK Tyr525/526 (Cat.#2711), anti-BTK (Cat.#8547), anti-phospho-BTK Tyr223 (Cat.# 87457), anti-MCL1 (Cat.# 94296), anti-BCLXL (Cat.# 2764), and anti-HSP90 (Cat.#4874). Anti-β-Actin (Cat.# SC-1616) was purchased from Santa Cruz Biotechnology (Dallas, TX) and anti-GAPDH (Cat.# MAB374) from EMD Millipore (Burlington, MA). HSP90, β-Actin, and GAPDH were used as loading controls.

### Cell treatment and immunoblotting

Cell lines were serum starved overnight followed by a 10-min treatment. Cells were washed in ice-cold PBS then lysed in 1X Cell Lysis Buffer (CST) supplemented with proteinase and phosphatase inhibitors cocktail (Roche). Pierce BCA Protein Assay was used to quantify the protein lysates (Thermo Fisher Scientific, Waltham, MA). For Immunoblotting, 15–25 μg of protein lysate was prepared in 2X or 4X Laemmeli’s sample buffer (Bio-Rad Laboratories, Hercules, CA) and heated for 5 min at 100 °C. Samples were separated on 8% sodium dodecyl sulfate polyacrylamide gel and transferred onto nitrocellulose membrane (Bio-Rad Laboratories, Hercules, CA). The membranes were blocked for 1 h in Blocker BLOTTO Blocking Buffer (Thermo Fisher Scientific, Waltham, MA) or 5% non-fat milk followed by overnight incubation at 4 °C with primary antibody followed by HRP-conjugated secondary antibodies and visualized with chemiluminescent Pierce ECL Substrate (Thermo Fisher Scientific, Waltham, MA) on X-ray films.

### Biochemical assay of recombinant kinases

Biochemical inhibition of FLT3-WT and FLT3-ITD fusion was measured using recombinant protein constructs of kinase domains (Reaction Biology). ARQ 531 was tested in a 10-point concentration mode with 3-fold serial dilutions starting at 1 μmol/L in the presence of 10 μmol/L ATP, and the IC_50_ was determined.

### Proliferation assay

A total of 5000–30,000 cells from the lines above were treated in a 96-well plate for 72 h. CellTiter 96 Aqueous MTS reagent (Promega, Madison, WI) was added to cells followed by a 3-h incubation at 37 °C, 5% CO2. The absorbance was measured at 490 nm using DTX 880 plate reader and the half maximal inhibitory concentration (IC_50_) was calculated using GraphPad Prism 7 (GraphPad Software, La Jolla, CA) and SAS/STAT software (SAS v9.4 for Windows, SAS Institute, Inc., Cary, NC). For the MOLM-13 resistant and the Ba/F3-FLT3-ITD/TKD cell lines, cells were treated for 72 h and cell viability was evaluated using MTT assay (Roche Diagnostics. Mannheim. Germany) according to the manufacturer’s instructions.

### Annexin/PI apoptosis assay

Cells were treated for 72 h then stained for 20 min in 1X Annexin Binding buffer (BD Biosciences, Franklin Lakes, NJ) containing Annexin V-FITC and Propidium Iodine (Leinco Technologies, Fenton, MO). Live and apoptotic cells were measured using FC 500 flow cytometer and analyzed on Kaluza software (Beckman Coulter, Pasadena, CA).

### Colony-Forming Unit-Granulocyte and Macrophage assay

Primary AML patient samples were treated and plated at 5000–10,000 cells in 1.1 ml methylcellulose supplemented with recombinant human stem cell factor, interleukin 3, granulocyte colony-stimulating factor, and granulocyte-macrophage colony-stimulating factor (MethoCult™, STEMCELL Technologies, Vancouver, Canada) per dish in duplicates. Colonies were counted after 14 days when using primary patient samples.

### In vivo efficacy studies

All experiments were conducted after approval of The Ohio State University Institutional Animal Care and Use Committee. Mice were randomized and enrolled for daily oral gavage (Q.D.P.O) treatment of vehicle or 50 mg/kg ARQ 531. Mice were treated until reaching early removal criteria (ERC) defined as 20% weight loss, lethargy, hind limb paralysis, moribund, anorexia, dehydration, hunched posture, severe diarrhea, breathing difficulties, or any abnormal neurological symptoms.

#### MOLM-13 xenograft AML model

A total of 10,000 luciferase expressing MOLM-13 cells [38] were engrafted via tail vein injection in NSG mice (The Jackson Laboratory, Bar Harbor, ME) for the single agent ARQ 531 study or NCG mice (Charles River Laboratories, Wilmington, MA) for the ARQ 531 + venetoclax (ARQ + Ven) combination study. For the first single agent ARQ 531 study, mice were treated 7 days post-engraftment. Due to the aggressiveness of the MOLM-13 model, mice were treated 4 days post-engraftment in the ARQ + Ven combination study. In both studies, mice were treated daily via oral gavage with vehicle (10% absolute alcohol, 10% Cremophor-EL, 80% saline) or 50 mg/kg ARQ 531, and 75 mg/kg venetoclax ± 50 mg/kg ARQ 531 for the combination study. Weekly in vivo imaging was performed using In Vivo Imaging System (IVIS) imager (PerkinElmer, Waltham, MA).

### Statistical analysis

**Fig. 1:**
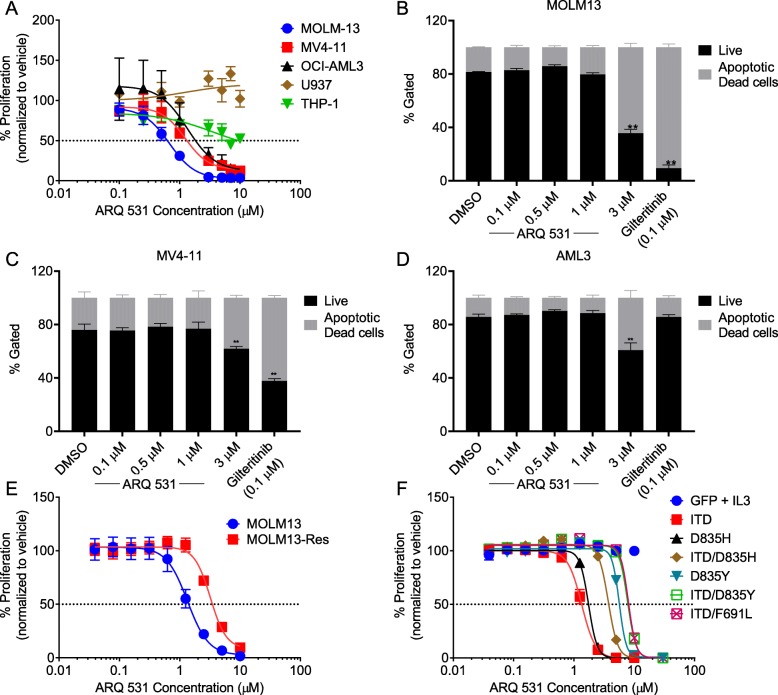
ARQ 531 demonstrates in vitro anti-leukemic effect. MTS proliferation assay for 72 h from three independent experiments using increasing concentrations of ARQ 531 treatment in MOLM-13, MV4-11, OCI-AML3, U937, and THP-1 AML cell lines (**a**). Representative of three replicates for Annexin-V/PI apoptosis assay for 72 h treatment of increasing concentrations of ARQ 531 or 50 nM of gilteritinib (Gilt.) in MOLM-13 (**b**), MV4-11 (**c**), and OCI-AML3 (**d**) cell lines. MTT proliferation assay for 72 h from two independent experiments using increasing of increasing concentrations of ARQ 531 in MOLM-13-resistant (MOLM13 Res) cell line (**e**) and Ba/F3 murine pro B cell line with FLT3-ITD or TKD or both FLT3-ITD-TKD mutations (**f**). **p ≤ 0.001

**Fig. 2:**
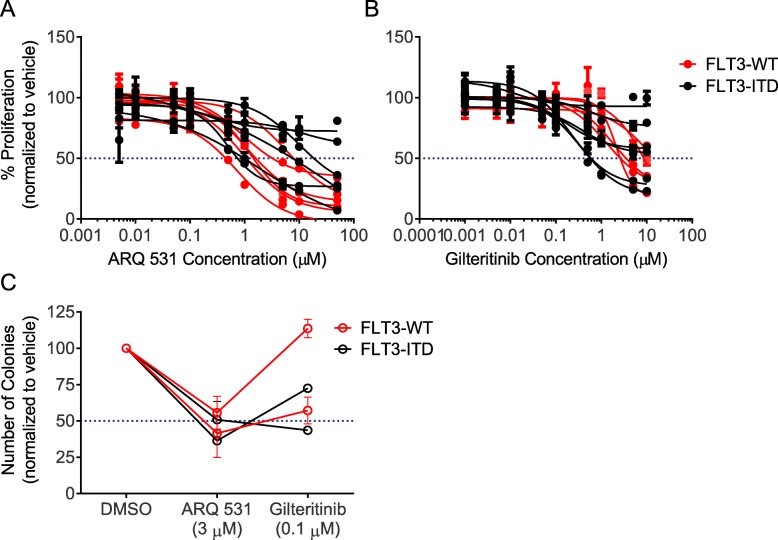
ARQ 531 demonstrates in vitro anti-leukemic effect in primary AML. MTS proliferation assay using a human stroma co-culture assay. Primary patient blasts (*n* = 11–12 pts) were co-cultured with HS5-GFP stroma cells and treated with increasing concentrations of ARQ 531 (**a**) or gilteritinib (**b**) for 96 h. FLT3 wild-type (WT) samples (red) and FLT3-ITD (black). Colony-Forming Unit-Granulocyte and Macrophage (CFU-GM) assay. Four primary patient blasts were treated with DMSO or three μM ARQ 531 or 0.1 μM gilteritinib (Gilt.) and then cultured in methylcellulose in duplicate dishes. Number of colonies were counted after 14 days (**c**)

For in vitro cytotoxicity experiments (Fig. 1b–d), repeated measures models were used to account for correlations among observations from the same experiment. The percentage of live cells was compared at each concentration with DMSO. Estimated IC_50_ with 95% confidence intervals (CI) were calculated using 4-parameter logistic models. Mixed effects models were used in experiments described in Additional file 1: Table S4 and Fig. 2c to account for correlations within a single patient. Outcome data were first log-transformed to stabilize variances and reduce skewness. For survival experiments (Figs. 5 and 6a), Kaplan-Meier curves were produced and group differences for Fig. 5 and assessed by the log-rank test and for Fig. 6a, *p* values have been adjusted for multiple comparisons using Holm’s procedure. *p* values from experiments with multiple endpoints were adjusted for multiple comparisons using either Holm’s procedure or Dunnett’s test (all comparisons against vehicle/DMSO). All analyses were performed using SAS/STAT software (SAS v9.4 for Windows, SAS Institute, Inc., Cary, NC).

## Results

### ARQ 531 is a novel multi-kinase inhibitor with anti-proliferative activity in AML

To investigate the activity of ARQ 531 in AML, we tested its ability to inhibit cell proliferation of AML cell lines and primary patient samples. ARQ 531 demonstrated anti-proliferative activity in FLT-ITD-mutated cell lines (MOLM-13 and MV4-11) in addition to the NPM1c^+^, DNMT3A-mutated OCI-AML3 cell line at low μM concentrations, while U937 and THP-1 cell lines were resistant to concentrations up to 10 μM (Additional file 2: Table S1 and Fig. 1a). To assess the direct effect of ARQ 531 on cell survival, we quantified the levels of live (Annexin−PI−), apoptotic (Annexin+PI−), and dead cells (Annexin+PI+) after ARQ 531 treatment. ARQ 531 induced cell apoptosis in MOLM-13, MV4-11, and OCI-AML3 cell lines while gilteritinib was cytotoxic in the FLT3-ITD cell lines only (Fig. 1b–d). These data suggest efficacy for ARQ 531 in both FLT3-ITD and FLT3-WT cells.

As one of the main resistance mechanisms to FLT3 inhibitors is the acquisition of TKD mutations, we tested the anti-proliferative activity of ARQ 531 in the MOLM-13 TKI-resistant (MOLM13-Res) cell line previously described in Zimmerman et al. [39]. In this model, MOLM-13 cells have gained a D835Y mutation via continuous exposure to the FLT3 inhibitor tandutinib [39]. Compared with the parental MOLM-13 cells, ARQ 531 shows a modest shift in IC_50_ (1.3 μM vs. 3.4 μM in MOLM-13 and MOLM13-Res cells, respectively; Additional file 3: Table S2 and Fig. 1e). Similarly, ARQ 531 retains anti-proliferative activity in the IL-3 independent Ba/F3 murine cell lines harboring either a FLT3-ITD mutation, a resistance conferring TKD mutation, or a combination of both (IC_50_ ranging from 1.2 to 7.6 μM; Additional file 3: Table S2 and Fig. 1f). The same murine cell lines have been previously shown to have a more robust response to midostaurin compared with the more specific FLT3 inhibitor quizartinib [39, 40], suggesting that a multi-kinase inhibitor such as ARQ 531 is likely to be effective in quizartinib-resistant samples.

To verify these results in primary cells, AML patient blasts (Additional file 4: Table S3) were co-cultured with HS5-GFP stroma cells for 96 h. Similar to gilteritinib, ARQ 531 demonstrated cytotoxicity in FLT-ITD samples in addition to FLT3-WT (Fig. 2a, b and Additional file 1: Tables S4). Moreover, reduced colony formation was observed at clinically attainable levels of ARQ 531 in both FLT3-ITD and FLT3-WT samples primary AML blasts (Fig. 2c and Additional file 5: Table S5) suggesting this agent has anti-clonogenic activity independent of FLT3 status.

### ARQ 531 modulates alterative oncogenic kinases

**Fig. 3:**
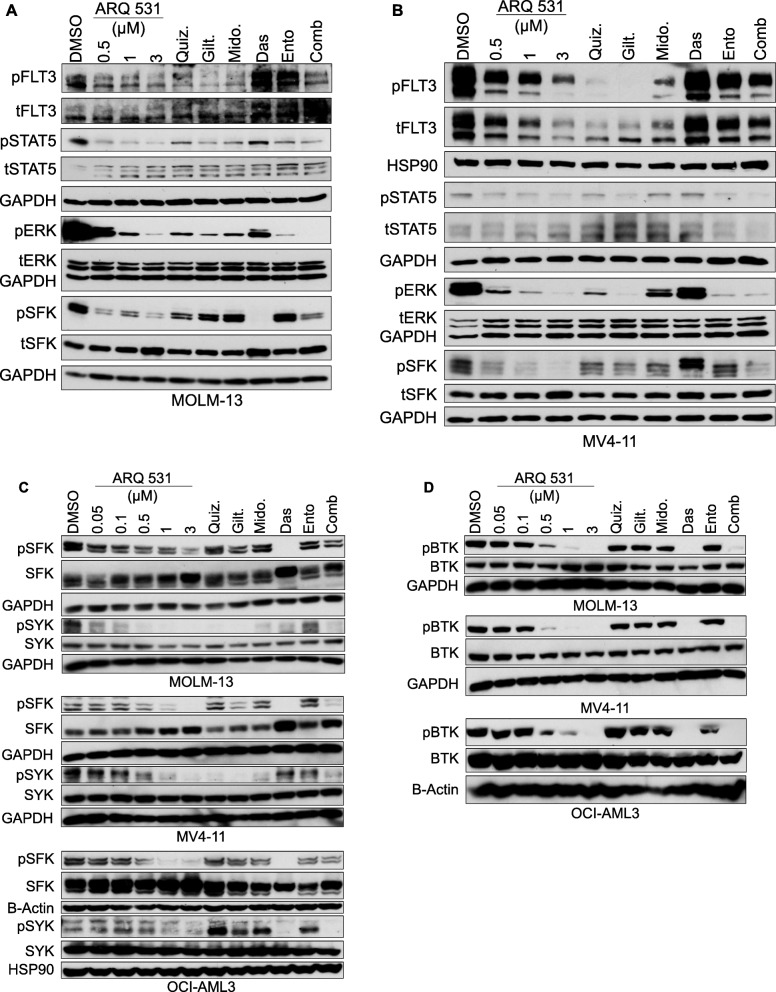
ARQ 531 modulates AML pro-survival kinases. Immunoblot analysis for MOLM-13, MV4-11, and OCI-AML3 AML cell lines (representative of 2-3 independent blots). All cell lines were serum starved overnight followed by 10-min treatment of five to 10 million cells with DMSO, increasing concentrations of ARQ 531 in comparison with 50 nM of quizartinib (Quiz.) or 50 nM gilteritinib (Gilt.), 0.1 μM midostaurin (Mido.), 0.5 μM dasatinib (Das.), 1 μM entospletinib (Ento.), or a combination of 1 μM ARQ 531 and Ento. Twenty to 25 μg of total protein lysate was loaded per lane. GAPDH, HSP90, or β-Actin were used as loading controls. **a, b** Modulation of phosphorylated SFK and downstream targets in FLT3-ITD cell lines MOLM-13 and MV-11, respectively. **c**, **d** Modulation of phosphorylated SYK and BTK in in all three cell lines

The equal cytotoxicity in FLT3-ITD and FLT3-WT cell lines suggests that the ARQ 531 mechanism of action is independent of FLT3 status. In vitro kinase profiling utilizing recombinant protein kinases revealed that ARQ 531 exhibited potent inhibition of SFK, including LCK, YES, HCK, LYNa, FGR, FYN, and FRK [36] in addition to BTK and FLT3 (WT and ITD; 82 nmol/L and 330 nmol/L, respectively). Thus, we tested the ability of ARQ 531 to downregulate the levels of phosphorylated SFK (Tyr416), phospho-FLT3 and downstream targets, phospho-STAT5, and phospho-ERK in AML cell lines including the FLT3-ITD MOLM-13 and MV4-11 and the FLT3-WT OCI-AML3 (Fig. 3). ARQ 531 inhibited the phosphorylated levels of SFK, FLT3, STAT5, and ERK at concentrations as low as 500 nM in the FLT3-ITD cell lines, MOLM-13 (Fig. 3a) and MV4-11 (Fig. 3b). This is consistent with other FLT3 inhibitors quizartinib (selective FLT3 inhibitor), gilteritinib (AXL/FLT3 inhibitor), and midostaurin (multi-kinase inhibitor), although the more selective inhibitors are effective against FLT3 at a lower concentration range (50–100 nM). Importantly, ARQ 531 potently inhibited the phosphorylation of Tyrosine 416 of the SFK in all three cell lines, MOLM-13, MV4-11, and OCI-AML3 (Fig. 3c) similar to the positive SFK inhibitor control, dasatinib, while the other FLT3 inhibitors had little to no effect on SFK phosphorylation (Fig. 3c) in the FLT3-WT OCI-AML3 cell line. This suggests a role for SFK modulation in the mechanism of ARQ 531 in AML cell lines, distinguishing it from other FLT3 or multi-kinase inhibitors. To confirm the functional inhibition of SFK by ARQ 531, we measured the level of phosphorylated SYK, as well as an integral downstream target of the SFK member, LYN. SYK is indispensable for myeloproliferative disease development and its transformation to AML [30] and a critical regulator of FLT3-ITD driven neoplasia due to its role in FLT3 transactivation [30]. In all three cell lines, ARQ 531 inhibited the phosphorylated levels of SYK at the tyrosine 525/526 activation loop site in the SYK kinase domain which is a marker for SYK function [41]. Interestingly, the selective FLT3 inhibitors, quizartinib and gilteritinib in addition to the multi-kinase inhibitor midostaurin also down-modulated SYK phosphorylation only in the FLT3-ITD cell lines, but not in the FLT3-WT OCI-AML3 cell line (Fig. 3c). This implies that the SYK down-modulation in the ITD cell lines is the result of abrogating FLT3-ITD-dependent SYK activation as previously suggested by Puissant et al. [30], whereas inhibition of SYK signaling can occur directly in the FLT3 WT OCI-AML3 cell line.

Several reports have shed light on the involvement of BTK in AML, especially in the context of FLT3-ITD mutation [32, 33, 42–44], and ARQ 531 potently inhibits BTK in the AML cell lines independent of FLT3 status (Fig. 3d). Although both ARQ 531 and ibrutinib both potently inhibit BTK phosphorylation, only ARQ 531 demonstrated in vitro anti-proliferative activity in MOLM-13 cell line (Additional file 6: Figure S2). Alternatively, the Syk inhibitor entospletinib while having no effect on BTK phosphorylation also potently inhibited proliferation in MOLM13 cells. These data suggest that the activity of ARQ 531 is attributed to the broad kinase inhibitory profile, beyond BTK.

Our data suggests the potential clinical benefit of ARQ 531 as a multi-kinase inhibitor targeting FLT3 upstream regulators, SFK and SYK and subsequently FLT3 and BTK. The effect does not appear to be FLT3 dependent, and further studies are warranted to identify mutational subsets most sensitive to ARQ 531.

### ARQ 531 activity is affected by SYK expression levels

We next determined if SYK inhibition is essential for the response to ARQ 531. We constitutively overexpressed the SYK-TEL fusion protein that has been previously utilized to study the role of SYK in response to therapeutic agents [30, 31]. The SYK-TEL construct maintains the SYK SH2 domains allowing the SYK and FLT3 interaction, and also contains a portion of the TEL/ETV6 transcription factor protein which results in enhanced FLT3 activation [30].

**Fig. 4:**
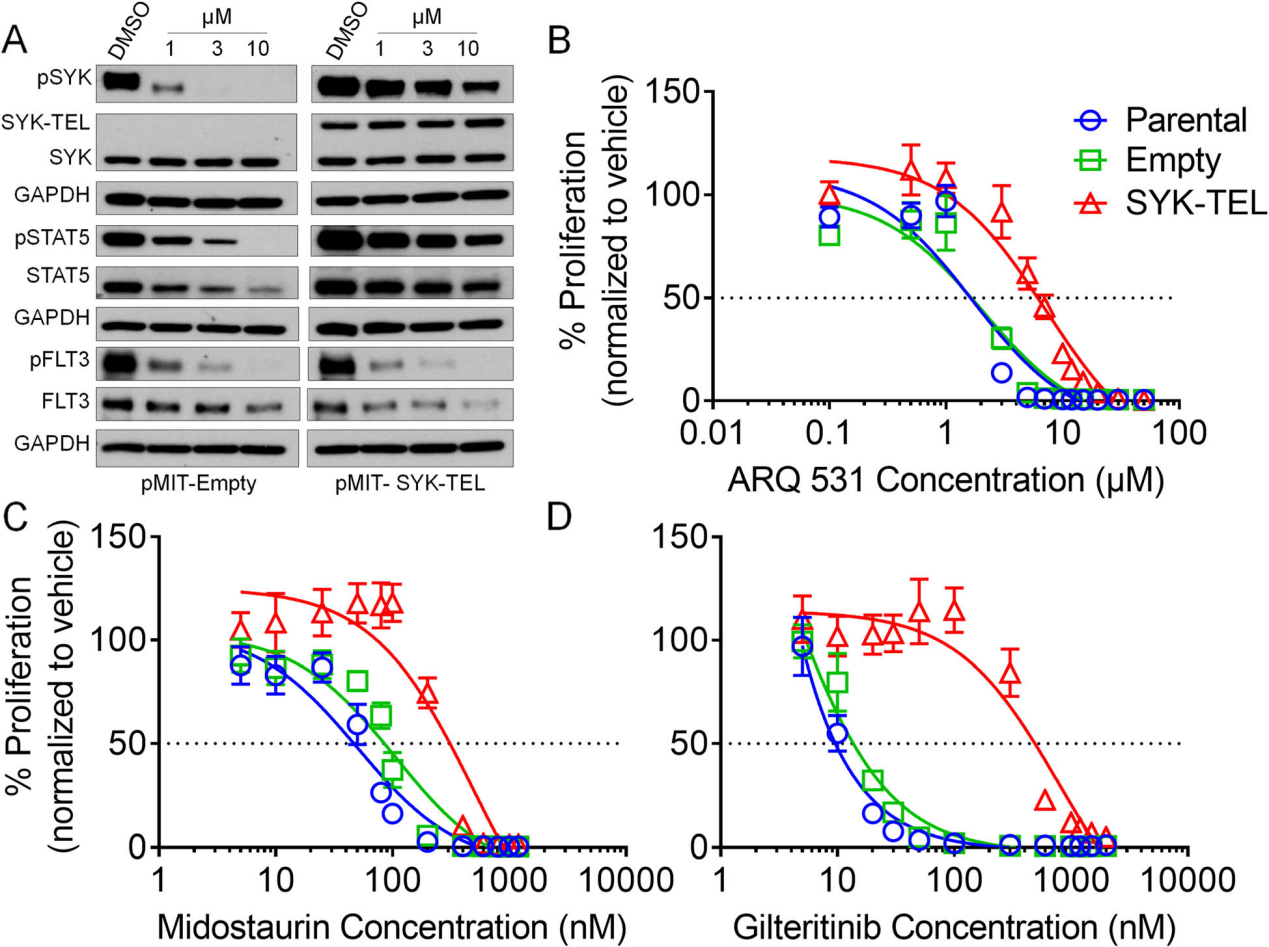
SYK levels effect ARQ 531 potency. Immunoblot analysis for pMIT-Empty or SYK-TEL overexpressing Ba/F3-FLT3-ITD cell lines treated with increasing concentrations of ARQ 531 (representative of three independent blots). All cell lines were serum starved overnight followed by a 3-h treatment of 10 million cells. Twenty micrograms of total protein lysate was loaded per lane. GAPDH was used as loading control. Images are from same immunoblot but showing different exposure time optimum to each cell line (**a**). MTS proliferation assay for 72 h from three independent experiments using SYK overexpressing Ba/F3-FLT3-ITD cell lines. Parental Ba/F3-FLT3-ITD, Ba/F3 FLT3-ITD expressing pMIT-Empty, or pMIT-SYK-TEL were treated for 72 h with increasing concentrations of ARQ 531 (**b**), midostaurin (**c**), or gilteritinib (**d**)

The Ba/F3 murine pro-B cell line expressing FLT3-ITD (IL-3 independent) [39] was transduced with pMIT-SYK-TEL or empty vector control to determine if the potency of ARQ 531 is reduced or abrogated with SYK overexpression. As expected, phosphorylated SYK was increased in the presence of SYK overexpression and we observed a reduced ability of ARQ 531 to decrease SYK and STAT5 phosphorylation in the pMIT-SYK-TEL expressing cell line compared with the pMIT-Empty control (Fig. 4a). Subsequently, there was more than twofold increase in the IC_50_ in the Ba/F3-FLT3-ITD pMIT-SYK-TEL cell line compared with the parental (Additional file 7: Table S6 and Fig. 4b) suggesting that the overexpression of SYK reduced ARQ 531 potency. Similarly, both midostaurin and gilteritinib exhibited the same pattern of reduced potency with SYK-TEL expression (Additional file 7: Table S6 and Fig. 4c, d). Altogether, ARQ 531 potency is reduced in the event of SYK overexpression suggesting that SYK is an important target for ARQ 531 activity.

### ARQ 531 exhibits modest in vivo anti-leukemic activity in an AML human xenograft mouse models as a single agent

**Fig. 5:**
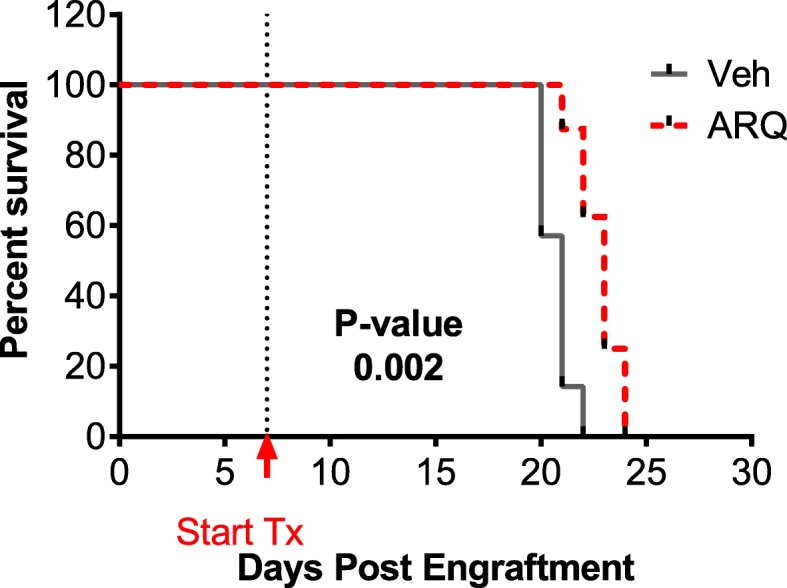
ARQ 531 exhibits in vivo anti-leukemic activity in AML. Kaplan–Meier survival curve in the MOLM-13 disseminated AML xenograft model. Mice were treated with vehicle (*N* = 7) or 50 mg/kg ARQ 531 Q.D.P.O (*N* = 8) at 7 days post engraftment until ERC

We next sought to investigate the in vivo activity of ARQ 531 in the previously described MOLM-13 xenograft model [38]. Seven days post-engraftment, mice were randomized to receive vehicle (*n* = 7) or 50 mg/kg ARQ 531 (*n* = 8) daily, a dose shown previously effective in TCL1 adoptive transfer mouse model of CLL [36]. Animals were monitored for ERC and overall survival (OS), with a subset (*n* = 3) injected with luciferin and imaged weekly for tumor burden (Additional file 8: Figure S1). Despite the aggressiveness of the MOLM-13 disseminated xenograft model, ARQ 531 demonstrated a modest yet significant median 2-day survival advantage (23 days vs. 21 days, log-rank *p* value = 0.002; Fig. 5 and Additional file 9: Table S7).

### BCL2 inhibition improves ARQ 531 in vivo anti-leukemic activity in AML

**Fig. 6:**
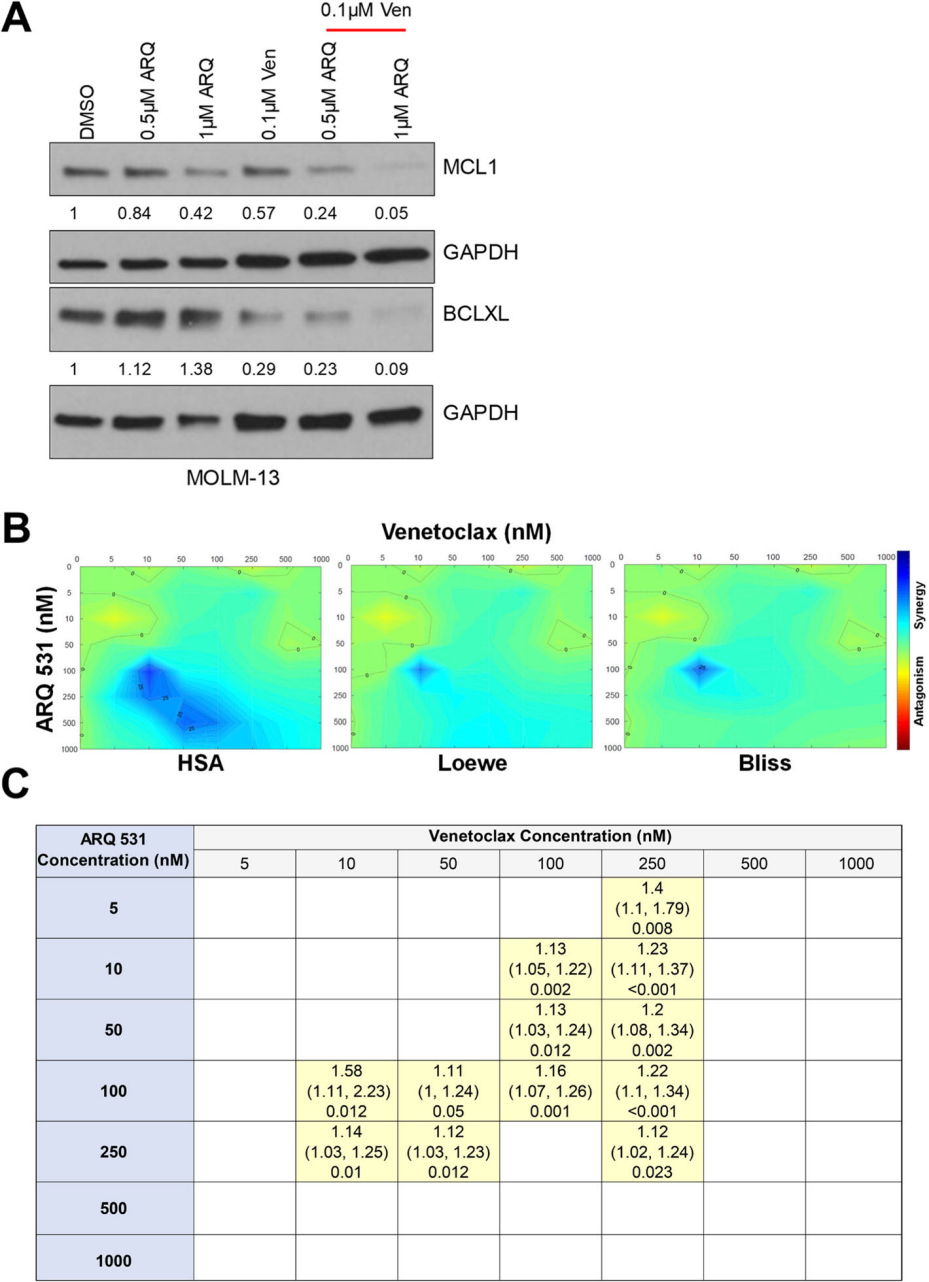
Immunoblot analysis for MCL1 modulation in MOLM-13 FLT3-ITD cell line (representative of two independent blots). Cells were serum starved and treated for 20 h with ARQ 531 alone or in combination with venetoclax. Twenty micrograms of total protein lysate was loaded per lane. GAPDH used as loading control. Band densitometry is done using ImageJ and quantified relative to GAPDH and DMSO treatment (**a**). In vitro analysis for additive or synergistic activity for ARQ 531 and venetoclax combination. Representative of three MTS proliferation assays. The MOLM-13 cell line was treated with increasing doses of ARQ 531 or venetoclax single agents or a combination of both for 72 h. Three biological replicates were analyzed using Combenefit software implementing the mathematical models for Highest Single Agent (HSA), Loewe, and Bliss (**b**). Table represent the concentrations with evidence of synergy (calculated ratio of proliferation with single agent alone vs. in combination > 1 via mixed effects model—see Additional file 11: Supplemental Information 1 for more details, *p* value ≤ 0.05) (**c**)

**Fig. 7:**
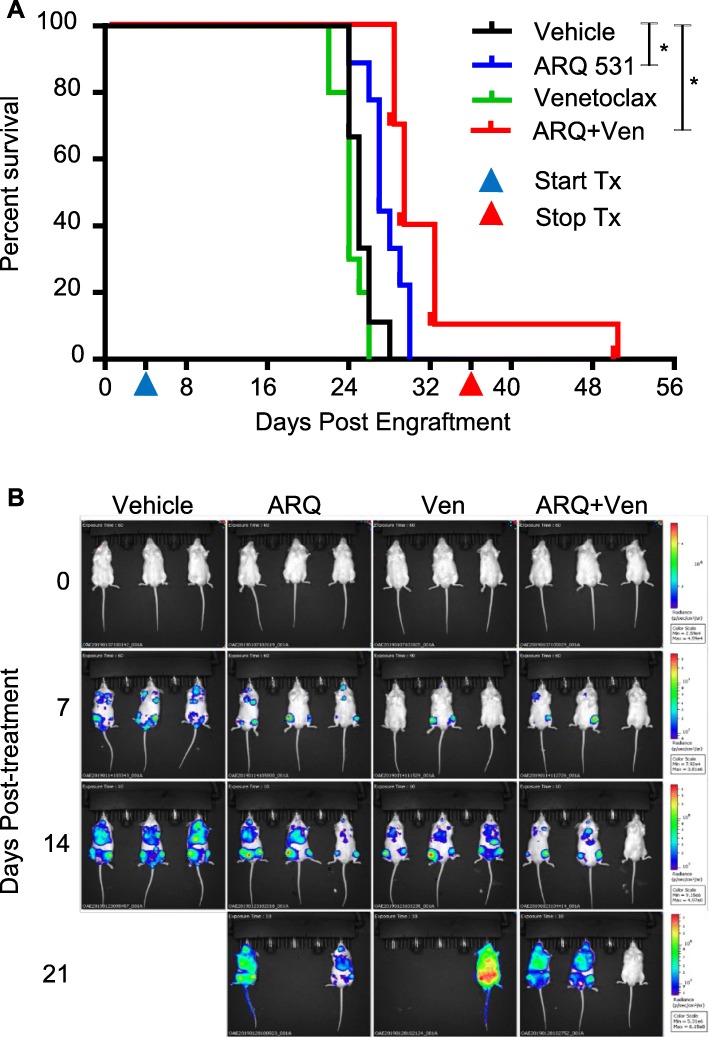
BCL2 inhibition improves ARQ 531 in vivo activity. **a** Kaplan–Meier survival curve in the MOLM-13 xenograft after mice were treated with vehicle (*N* = 9) or 50 mg/kg ARQ 531 Q.D.P.O (*N* = 9) or 75 mg/kg venetoclax Q.D.P.O (*N* = 10) or 50 mg/kg ARQ 531 and 75 mg/kg venetoclax combination Q.D.P.O (*N* = 10) at 4 days post engraftment until ERC. **b** Noninvasive serial whole body bioluminescence imaging of ventral view to monitor leukemia progression and regression post treatment. Mice were injected with 150 mg/kg Firefly D-Luciferin for evaluation of tumor burden as determined by whole body bioluminescence imaging using IVIS imager

BCL-2 inhibition using venetoclax has shown modest activity as a monotherapy in AML [45], and venetoclax resistance has been reported especially in the context of MCL-1 upregulation [46]. Therefore, combination approaches aimed to synergistically enhance venetoclax activity and overcome resistance have been explored, such as combinations with a BRD4 inhibitor [47], an MCL-1 inhibitor [45–47], or an MDM2 antagonist [48]. We examined if ARQ 531, to its strong inhibition of phosphorylated ERK which is essential for MCL-1 expression [49], is synergistic with venetoclax. In the MOLM-13 FLT-ITD cell line, we observed MCL1 downregulation with ARQ 531 treatment and a stronger inhibition when combined with venetoclax (Fig. 6a). Next, we determined potential additivity or synergy for the ARQ 531 + venetoclax (ARQ + Ven) combination compared with ARQ 531 monotherapy using the Combenefit software [50], which implements the classical mathematical models Highest Single Agent (HSA), Loewe, and the Bliss (Fig. 6b). As an additional check, we determined if the estimated decrease in proliferation in the presence of ARQ + Ven was greater than the decrease in proliferation with ARQ alone added to the decrease in proliferation with Ven alone (that is, the additive effect of ARQ, Ven) via separate mixed effects models for each dose combination. We considered all combinations with *p* < 0.05 as potential candidates for synergy (Fig. 6c and Additional file 11). Collectively, our in vitro analysis suggests enhanced activity for ARQ 531 in combination with the BCL-2 inhibitor, venetoclax. Subsequently, we tested if the ARQ + Ven combination enhanced ARQ 531 in vivo again using the aggressive MOLM-13 disseminated AML xenograft model. We observed a prolonged survival in the ARQ + Ven cohort in comparison with either agents alone (ARQ + Ven, 29 days; ARQ 531, 27 days; venetoclax, 24 days; vehicle, 25 days; overall log-rank test *p* value < 0.001; Fig. 7a and Additional file 9: Table S7) and reduced tumor burden (Fig. 7b, Additional file 10: Figure S3). Altogether, ARQ + Ven exhibited an increased survival advantage compared with ARQ 531 single therapy in an aggressive AML xenograft model.

## Discussion

Despite the approval of many new agents, AML treatment remains a significant challenge. Herein, we demonstrate the preclinical activity of the multi kinase inhibitor ARQ 531 in AML. While ARQ 531 is currently under clinical evaluation in CLL, our data also supports a broad anti-proliferative and cytotoxic activity in both FLT-ITD and FLT3-WT AML cell lines and primary AML patient samples. ARQ 531 inhibited the phosphorylation of FLT3, STAT5, and ERK similar to FLT3 inhibitors (quizartinib, gilteritinib, and midostaurin). Additionally, it strongly inhibited SFK phosphorylation suggesting the importance of modulating SFK or a downstream target of SFK in AML and potentially explaining the activity of ARQ 531 in FLT3-WT cells. To confirm the role of SFK inhibition in response to ARQ 531, we examined SYK phosphorylation, a critical SFK downstream target. Indeed, phosphorylated SYK levels were reduced by ARQ 531, and SYK constitutive overexpression caused a shift in ARQ 531 IC_50_, implicating a role for SYK modulation in ARQ 531 activity in AML cell lines. We extended these mechanistic signaling studies to demonstrate in vivo activity with daily oral administration of 50 mg/kg ARQ 531 in the aggressive MOLM-13 xenograft model and demonstrated synergy of this with the BCL2 antagonist venetoclax which recently gained accelerated FDA approval as combination therapy in previously untreated AML. Collectively, these data provide support for extended development of ARQ 531 in myeloid malignancies, particularly in combination with other agents for subsets of disease dependent upon SFK kinase signaling.

ARQ 531 also potently inhibits BTK and previously demonstrated a remarkable survival advantage in two immunocompetent adoptive transfer mouse models of CLL, Eμ-TCL1, and Eμ-MYC/TCL1 when compared with ibrutinib [36] resulting in a phase 1 dose escalation clinical trial in relapsed or refractory CLL [37]. In this ongoing trial, concentration steady-state levels of ARQ 531 exceeding 1 μM have been observed with escalation continuing. An explanation for the modest in vivo activity in AML models could be the fact that ARQ 531 activity against AML potentially needs a constant steady-state plasma concentration (*C*_ss_) in the μM range unlike in CLL where BTK phosphorylation inhibition is achieved in nM range. Additionally, the PK profile for ARQ 531 is very different between primates and rodents. In *Macaca fascicularis* cynomolgus monkeys, a single oral dose of 10 mg/kg demonstrated favorable PK and oral bioavailability achieving maximum concentration (*C*_max_) of 9 μM and 72% bioavailability. Due to the absence of elimination phase, the half-life (*t*_1/2_) was not calculated where 75% of peak concentration was present at 24 h, suggesting that ARQ 531 has a long plasma half-life [36]. The completion of the ongoing phase 1 dose escalation clinical trials in relapsed or refractory CLL patients [37] will be crucial to determine the achievable *C*_ss_ plasma concentration in patients and the potential use of ARQ 531 in AML. At the previous cleared 65 mg daily dose of ARQ 531, the mean *C*_max_ of ~ 3 μM was obtained [51] suggesting effective dose levels in AML may be achievable.

To date, ARQ 531 demonstrated a manageable safety profile with no reported treatment emergent adverse events in the dose escalation study [37]. This is consistent with the lack of toxicity in both our study and those previously performed in CLL xenograft mouse models [36], suggesting ARQ 531 is well tolerated and can potentially be used in combination with other therapeutics. Venetoclax is being actively investigated in combination with various drugs in AML [45–48]. We saw in vitro synergistic activity for ARQ 531 and venetoclax and a survival advantage in the ARQ + Ven cohort (compared with single agents) suggesting an additive benefit to the ARQ 531 and venetoclax combination. Because the therapeutic efficacy with ARQ 531 may be best recognized in combination with other targeted agents, efforts to utilize CRISPR-Cas9 screens to identify synthetic lethality with ARQ 531 will support further drug combinations or identify additional sensitive patient populations. These studies offer the potential to also identify the important targets of ARQ 531 in myeloid versus lymphoid cells. Future in vivo studies using patient-derived xenograft (PDX) models representing different AML mutation subtypes will further validate the benefit of ARQ 531 as a therapeutic option in AML.

## Conclusions

In summary, we present the first preclinical assessment of the multi-kinase inhibitor ARQ 531 in AML. We report in vitro anti-leukemic activity independent of FLT3 mutation status and modest in vivo activity that might be more evident in larger animal models based upon observed interspecies pharmacokinetic differences. Further optimization studies are needed to identify an in vivo dose and schedule capable of achieving a sustained, favorable PK profile in mice for its evaluation in AML. Collectively, our findings suggest that ARQ 531 could have a role in AML therapy especially with the high plasma concentration reached in patients, but likely will be most impactful if administered in rational combinations with drugs that effectively synergize with ARQ 531.

## Supplementary information

**Additional file 1: Table S4.** ARQ 531 and Gilteritinib efficacy in patient samples.

**Additional file 2: Table S1.** Estimated absolute IC50 based on a 4-parameter logistic model in AML cell lines.

**Additional file 3: Table S2.** Estimated absolute IC50 based on a 4-parameter logistic model in MOLM-13 resistant and Ba/F3 FLT3-ITD cell lines.

**Additional file 4: Table S3.** Patient samples mutation information.

**Additional file 5: Table S5.** Comparison of colony formation in patient samples.

**Additional file 6: Figure S2.** Immunoblot analysis for ARQ 531 comparison with ibrutinib irreversible BTK inhibitor and entospletinib SYK inhibitor.

**Additional file 7: Table S6.** Estimated IC50 calculations in SYK overexpressing cell lines.

**Additional file 8: Figure S1.** Bioluminescence imaging for ARQ 531 in vivo monotherapy.

**Additional file 9: Table S7.** ARQ 531 in vivo activity in MOLM-13 disseminated AML xenograft model.

**Additional file 10: Figure S3.** Bioluminescence imaging for ARQ 531 in vivo combination therapy.

**Additional file 11.** Supplementary Information 1.

## Data Availability

Data sharing is not applicable to this article as no datasets were generated or analyzed during the current study.

## Abbreviations

AML: Acute myeloid leukemia
ARQ 531: ArQule 531
BTK: Bruton’s tyrosine kinase
CFU-GM: Colony-Forming Unit-Granulocyte and Macrophage
CI: Confidence intervals
CLL: Chronic lymphocytic leukemia
C_max_: Maximum concentration
C_ss_: Steady state plasma concentration
ERC: Early removal criteria
FDA: Federal Drug Administration
FLT3: Fms related tyrosine kinase 3
IC_50_: Half maximal inhibitory concentration
ITD: Internal tandem duplication
IVIS: In Vivo Imaging System
OS: Overall survival
PK: Pharmacokinetic
Q.D.P.O: Daily oral gavage
SFK: Src FAMILY kinases
SYK: Spleen tyrosine kinase
t_1/2_: Half-life
TKD: Tyrosine kinase domain
