# Supplementary material for: Preclinical efficacy for a novel tyrosine kinase inhibitor, ArQule 531 against acute myeloid leukemia
Source: J Hematol Oncol. 2020 Jan 28;13:8. doi: 10.1186/s13045-019-0821-7 (PMC6988309; doi:10.1186/s13045-019-0821-7)
Supplement: Supplementary file 1 — Additional file 1: Table S4. ARQ 531 and Gilteritinib efficacy in patient samples. [file 13045_2019_821_MOESM1_ESM.pdf]

Supplementary Table S4. ARQ 531 and Gilteritinib efficacy in primary patient's samples

ARQ 531			
**Concentration (μM) Comparison**	**Estimated Ratio**	**95% CI**	**P-value**
0.005 Versus Vehicle	0.91	(0.79, 1.05)	0.438
0.01 Versus Vehicle	0.95	(0.82, 1.1)	0.936
0.05 Versus Vehicle	0.92	(0.79, 1.06)	0.489
0.1 Versus Vehicle	0.9	(0.78, 1.04)	0.259
0.5 Versus Vehicle	0.69	(0.6, 0.8)	<.001
1 Versus Vehicle	0.58	(0.5, 0.68)	<.001
5 Versus Vehicle	0.34	(0.25, 0.48)	<.001
10 Versus Vehicle	0.28	(0.15, 0.52)	<.001
50 Versus Vehicle	0.09	(0, 2.04)	0.158
**Gilteritinib**			
**Concentration (μM) Comparison**	**Estimated Ratio**	**95% CI**	**P-value**
0.005 vs. 0 (DMSO)	1.02	(0.89, 1.17)	>.999
0.01 vs. 0	1.05	(0.92, 1.21)	0.926
0.05 vs. 0	1.06	(0.92, 1.21)	0.889
0.1 vs. 0	1.02	(0.89, 1.17)	>.999
0.5 vs. 0	0.99	(0.86, 1.13)	>.999
1 vs. 0	0.87	(0.76, 1)	0.05
5 vs. 0	0.78	(0.67, 0.9)	<.001
10 vs. 0	0.56	(0.47, 0.66)	<.001
50 vs. 0	0.54	(0.3, 0.98)	0.041
