# Supplementary material for: Preclinical efficacy for a novel tyrosine kinase inhibitor, ArQule 531 against acute myeloid leukemia
Source: J Hematol Oncol. 2020 Jan 28;13:8. doi: 10.1186/s13045-019-0821-7 (PMC6988309; doi:10.1186/s13045-019-0821-7)
Supplement: Supplementary file 2 — Additional file 2: Table S1. Estimated absolute IC50 based on a 4-parameter logistic model in AML cell lines. [file 13045_2019_821_MOESM2_ESM.pdf]

Supplementary Table S1. Estimated absolute IC_{50} based on a 4-parameter logistic model.

Cell Line	Estimated absolute µM IC_{50} (95% CI)	Mutations
MOLM-13	0.69 (0.59, 0.79)	NF1, FLT3, BIRC6, CBL
MV4-11	1.45 (1.04, 1.85)	TP53, FLT3
OCI-AML3	1.51 (0.60, 2.43)	SPI1, DNMT3A, NPM1, NRAS
THP-1	Unable to calculate IC_{50}	TP73, NRAS, SMARCA2
U937	Unable to calculate IC_{50}	ETV6, JAK3, MED12, PTEN, PTPN11, WT1
