# Supplementary material for: Preclinical efficacy for a novel tyrosine kinase inhibitor, ArQule 531 against acute myeloid leukemia
Source: J Hematol Oncol. 2020 Jan 28;13:8. doi: 10.1186/s13045-019-0821-7 (PMC6988309; doi:10.1186/s13045-019-0821-7)
Supplement: Supplementary file 3 — Additional file 3: Table S2. Estimated absolute IC50 based on a 4-parameter logistic model in MOLM-13 resistant and Ba/F3 FLT3-ITD cell lines. [file 13045_2019_821_MOESM3_ESM.pdf]

Supplementary Table S2. Estimated absolute IC_{50} based on a 4-parameter logistic model.

**Cell line/Mutation**	**Estimated absolute µM IC_{50} and 95% CI**
MOLM13	1.33 (1.26, 1.41)
MOLM13-Res	3.42 (3.25, 3.58)
Ba/F3 GFP+IL3	Unable to calculate IC_{50}
Ba/F3 FLT3-ITD	1.21 (1.16, 1.26)
Ba/F3 FLT3-D835H	1.74 (1.64, 1.85)
Ba/F3 FLT3-D835Y	6.11 (5.39, 6.83)
Ba/F3 FLT3-ITD/D835H	3.76 (3.5, 4.03)
Ba/F3 FLT3-ITD/D835Y	7.55 (6.99, 8.12)
Ba/F3 FLT3-ITD/F691L	7.12 (6.44, 7.81)
