# Supplementary material for: Preclinical efficacy for a novel tyrosine kinase inhibitor, ArQule 531 against acute myeloid leukemia
Source: J Hematol Oncol. 2020 Jan 28;13:8. doi: 10.1186/s13045-019-0821-7 (PMC6988309; doi:10.1186/s13045-019-0821-7)
Supplement: Supplementary file 4 — Additional file 4: Table S3. Patient samples mutation information. [file 13045_2019_821_MOESM4_ESM.pdf]

Supplementary Table S3. Primary patient samples information

Sample ID	Sample Type	FLT3 Status	Additional Mutations
U-11-1271	Bone Marrow	WT	
U-11-1363	Bone Marrow	WT	
U-12-1567	Bone Marrow	WT	
U-14-1077	Apheresis	WT	BRINP3, NPM1
U-15-0251	Apheresis	WT	
U-15-2962	Apheresis	WT	FLT3, BCOR
U-07-0614	Bone Marrow	ITD	
U-09-1270	Bone Marrow	ITD	
U-09-1409	Bone Marrow	ITD	
U-10-0874	Bone Marrow	ITD	
U-14-2181	Apheresis	ITD	FLT3, NPM1, RAD21, TET2
U-15-3590	Apheresis	ITD	BCORL1, TET2, NPM1, KMT2A, DNMT3A
AML1	Apheresis	WT	NRAS, WT1
