# Supplementary material for: Preclinical efficacy for a novel tyrosine kinase inhibitor, ArQule 531 against acute myeloid leukemia
Source: J Hematol Oncol. 2020 Jan 28;13:8. doi: 10.1186/s13045-019-0821-7 (PMC6988309; doi:10.1186/s13045-019-0821-7)
Supplement: Supplementary file 5 — Additional file 5: Table S5. Comparison of colony formation in patient samples. [file 13045_2019_821_MOESM5_ESM.pdf]

Supplementary Table S5. Comparison of Colony Formation in Primary Patient Samples

**Comparison**	**Estimated ratio**	**95% CI**	**p-value**
ARQ 531 (3 µM) vs. DMSO	0.45	(0.33, 0.6)	<.001
Gilteritinib (0.1 µM) vs. DMSO	0.67	(0.5, 0.9)	0.009
