# Supplementary material for: Preclinical efficacy for a novel tyrosine kinase inhibitor, ArQule 531 against acute myeloid leukemia
Source: J Hematol Oncol. 2020 Jan 28;13:8. doi: 10.1186/s13045-019-0821-7 (PMC6988309; doi:10.1186/s13045-019-0821-7)

Supplementary Figure S2. Immunoblot analysis for MOLM-13 FLT3-ITD cell line treated for 1 hour with 1 μM of ARQ 531 multi-kinase inhibitor, ibrutinib irreversible BTK inhibitor, and entospletinib SYK inhibitor **(A)**. 72 hours MTS proliferation assay using MOLM-13 cell line treated with 1 μM of ARQ 531, ibrutinib (1 hour washout), or entospletinib **(B)**.

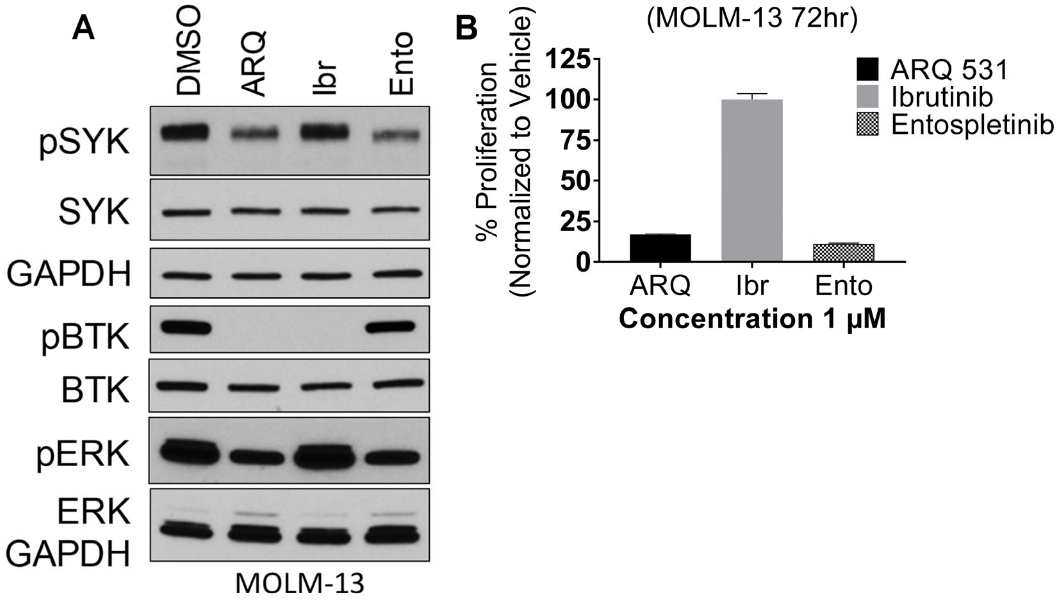

Supplement: Supplementary file 6 — Additional file 6: Figure S2. Immunoblot analysis for ARQ 531 comparison with ibrutinib irreversible BTK inhibitor and entospletinib SYK inhibitor. [file 13045_2019_821_MOESM6_ESM.pdf]
