# Supplementary material for: Preclinical efficacy for a novel tyrosine kinase inhibitor, ArQule 531 against acute myeloid leukemia
Source: J Hematol Oncol. 2020 Jan 28;13:8. doi: 10.1186/s13045-019-0821-7 (PMC6988309; doi:10.1186/s13045-019-0821-7)
Supplement: Supplementary file 7 — Additional file 7: Table S6. Estimated IC50 calculations in SYK overexpressing cell lines. [file 13045_2019_821_MOESM7_ESM.pdf]

Supplementary Table S6. Estimated IC_{50} calculations in SYK overexpressing cell lines

Drug	Cell Line	Experiment 1	Experiment 2	Overall
**ARQ-531**	Ba/F3 FLT3-ITD Parental	2.71	2.53	2.71
		(2.6, 2.83)	(1.71, 3.34)	(2.6, 2.82)
	Ba/F3 FLT3-ITD pMIT-Empty	4.24	2.7	3.48
		(4.07, 4.42)	(2.53, 2.88)	(3.35, 3.6)
	Ba/F3 FLT3-ITD pMIT SYK-TEL	11.02	5.94	6.61
		(10.13, 11.91)	(5.6, 6.29)	(6.29, 6.92)
**Gilteritinib**	Ba/F3 FLT3-ITD Parental	20.46	9.49	16.81
		(19.54, 21.37)	(8.19, 10.78)	(16.08, 17.55)
	Ba/F3 FLT3-ITD pMIT-Empty	47.17	15.11	24.56
		(45.39, 48.94)	(13.97, 16.26)	(23.61, 25.51)
	Ba/F3 FLT3-ITD pMIT SYK-TEL	650.55	415.42	456.48
		(572.74, 728.37)	(379.62, 451.21)	(424.51, 488.46)
**Midostaurin**	Ba/F3 FLT3-ITD Parental	95.38	63.32	76.25
		(91.73, 99.03)	(60.32, 66.32)	(73.97, 78.53)
	Ba/F3 FLT3-ITD pMIT-Empty	197.95	95.32	96.29
		(162.87, 233.02)	(91.9, 98.74)	(92.94, 99.64)
	Ba/F3 FLT3-ITD pMIT SYK-TEL	403.64	229.56	280.84
		(378.92, 428.37)	(213.58, 245.54)	(267.64, 294.03)
