# Supplementary material for: Preclinical efficacy for a novel tyrosine kinase inhibitor, ArQule 531 against acute myeloid leukemia
Source: J Hematol Oncol. 2020 Jan 28;13:8. doi: 10.1186/s13045-019-0821-7 (PMC6988309; doi:10.1186/s13045-019-0821-7)

Supplementary Figure S1. Mice were injected with 150 mg/kg Firefly D-Luciferin for the evaluation of ventral and dorsal view tumor burden as determined by whole body bioluminescence imaging using IVIS imager.

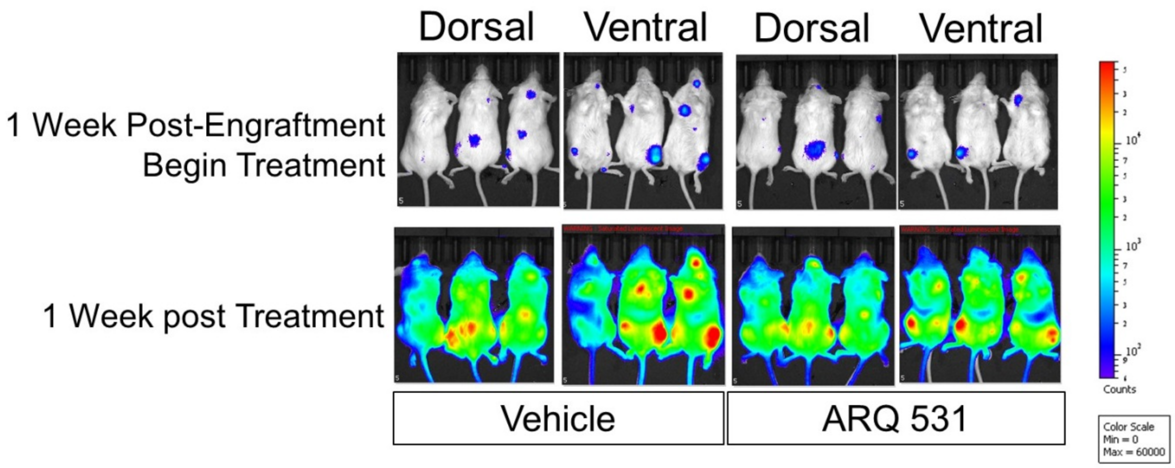

Supplement: Supplementary file 8 — Additional file 8: Figure S1. Bioluminescence imaging for ARQ 531 in vivo monotherapy. [file 13045_2019_821_MOESM8_ESM.pdf]
