# Supplementary material for: Preclinical efficacy for a novel tyrosine kinase inhibitor, ArQule 531 against acute myeloid leukemia
Source: J Hematol Oncol. 2020 Jan 28;13:8. doi: 10.1186/s13045-019-0821-7 (PMC6988309; doi:10.1186/s13045-019-0821-7)
Supplement: Supplementary file 9 — Additional file 9: Table S7. ARQ 531 in vivo activity in MOLM-13 disseminated AML xenograft model. [file 13045_2019_821_MOESM9_ESM.pdf]

Supplementary Table S7. ARQ 531 *in vivo* activity in MOLM-13 disseminated AML xenograft model.

ARQ 531 Monotherapy					
Group	N original	N engraft	N died	Estimated Median Survival Time (Days)	95% CI
Vehicle	7	7	7	21	(20, 21)
ARQ 531	8	8	8	23	(21, 24)
Log-rank p-value = 0.002					
ARQ 531 + Venetoclax Combination					
Group	N original	N engraft	N died	Estimated median survival time (days)	95% CI
Vehicle	10	9	9	25	(24, 26)
ARQ 531	10	9	9	27	(24, 30)
Venetoclax	10	10	10	24	(22, 25)
ARQ 531 + Venetoclax	10	10	10	29	(28, 32)
Overall log-rank test p-value <0.001					
