# Supplementary material for: Preclinical efficacy for a novel tyrosine kinase inhibitor, ArQule 531 against acute myeloid leukemia
Source: J Hematol Oncol. 2020 Jan 28;13:8. doi: 10.1186/s13045-019-0821-7 (PMC6988309; doi:10.1186/s13045-019-0821-7)
Supplement: Supplementary file 11 — Additional file 11. Supplementary Information 1. [file 13045_2019_821_MOESM11_ESM.pdf]

Supplementary Information

Supplementary Information 1

DMSO = observed proliferation with DMSO
ARQ = observed proliferation with ARQ 531
Ven = observed proliferation with Venetoclax
Combo = observed proliferation with the combination of ARQ 531 + Venetoclax

For the proliferation experiments, we define synergy as follows:

(Combo – DMSO) < (ARQ – DMSO) + (Ven – DMSO)

That is, in the presence of synergy we expect the reduction in proliferation with the combination vs. DMSO (Combo – DMSO) to be greater than the sum of the reductions seen individually with ARQ and Venetoclax alone [(ARQ – DMSO) + (Ven –DMSO)]. Note that the greater the reduction in proliferation, the *smaller* the proliferation relative to DMSO.

Before fitting a model, we applied a natural log transform to the data. The following abbreviations are used below:

A = ln(DMSO)
B =ln(ARQ)
C =ln(Ven)
D =ln(Combo)

Therefore, to show synergy, we would like to see

$(D - A) < (B - A) + (C - A)$

$\Rightarrow$ $-(D - A) > -[(B - A) + (C - A)]$ (multiply both sides by -1, reverse the direction of the inequality)

$\Rightarrow$ $(A - D) - [(A - B) + (A - C)] > 0$

$\Rightarrow$ $C - D + B - A > 0$

$\Rightarrow$ $(B + C) - (A + D) > 0$

$\Rightarrow$ $e^{(B+C) - (A+D)} > e^0$ (exponentiate both sides)

$\Rightarrow$ $(e^B e^C)/(e^A e^D) > 1$

$\Rightarrow$ $(e^B/e^A) \times (e^C/e^D) > 1$

$\Rightarrow$ $(e^{\ln(ARQ)}/e^{\ln(DMSO)}) \times (e^{\ln(Ven)}/e^{\ln(Combo)}) > 1$

$\Rightarrow$ (ARQ/DMSO) × (Ven/Combo) > 1

$\Rightarrow$ (ARQ/DMSO) /(Combo/Ven) > 1

$\Rightarrow$ (Effect of ARQ vs. DMSO) / (Relative effect of ARQ in combination) > 1

⇒ The ratio of proliferation effect with ARQ alone vs. in combination with Ven > 1

That is, in the presence of synergy we would expect to see more proliferation (less reduction) with ARQ alone than what we would see in combination with Venetoclax. By this same logic, a ratio < 1 implies antagonism.

Therefore, to test for synergy we fit a model using the natural log-transformed data for a fixed dose of ARQ, Ven and exponentiated the estimate of ln(Ven) – ln(combination) + ln(ARQ) – ln(DMSO) to obtain the ratio shown above. If the ratio was significantly greater than 1 ($p<0.05$) we considered this combination a potential candidate for synergy.
